# Achieving Pressure Consistency in Mechanochemical Simulations of Chemical Reactions Under Pressure

**DOI:** 10.1002/jcc.70024

**Published:** 2025-01-26

**Authors:** Jonas Bentrup, Rahel Weiß, Felix Zeller, Tim Neudecker

**Affiliations:** ^1^ Institute for Physical and Theoretical Chemistry University of Bremen Bremen Germany; ^2^ Bremen Center for Computational Materials Science University of Bremen Bremen Germany; ^3^ MAPEX Center for Materials and Processes University of Bremen Bremen Germany

## Abstract

The eXtended Hydrostatic Compression Force Field (X‐HCFF) is a mechanochemical approach in which a cavity is used to exert hydrostatic pressure on a target system. The cavity used in this method is set up to represent the van der Waals (VDW) surface of the system by joining spheres sized according to the respective atomic VDW radii. The size of this surface can be varied via a scaling factor, and it can be shown that the compression forces exerted in X‐HCFF in its current implementation depend on this factor. To address this dependency, we have developed a rescaling formalism for the applied forces, allowing us to drastically reduce the dependency of the compression forces on the chosen scaling factor. Independency from the scaling factor is important, as the scaling of the VDW spheres is often used to ensure an overlap of cavities in supramolecular complexes, which is necessary for the simulation of chemical reactions. Our rescaling formalism reduces the empiricism of the X‐HCFF approach and boosts its applicability in the field of computational high‐pressure chemistry.

## Graphical abstract

The eXtended Hydrostatic Compression Force Field (X‐HCFF) applies hydrostatic pressure in electronic structure calculations via a cavity representing the van der Waals surface of a molecular system. The surface size, adjustable by a scaling factor, influences the compression forces, molecular geometries, and other quantities calculated with X‐HCFF. To minimize this dependency, a rescaling formalism was developed, which improves pressure consistency regardless of the scaling factor. The rescaling formalism reduces empiricism and broadens the applicability of X‐HCFF in computational high‐pressure chemistry.

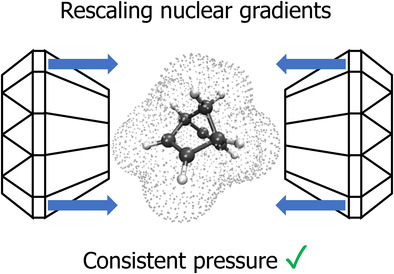

## 1 Introduction

It has long been known that the use of high pressures allows the manipulation of the reaction equilibrium according to le chatelier's principle, such as in the Haber‐Bosch synthesis of ammonia [1, 2]. The invention of technologies like diamond anvil cells [3] and shock wave generators [4] made pressures > 100 GPa available in today's chemical laboratories. These pressures pave the way for the synthesis of otherwise inaccessible compounds [5] and can lead to changes in molecular properties such as conductivity [6], dipole moment [7], color [8] and many more [9].

A challenging yet important task is the development of computational methods that allow the simulation of molecules or atoms under pressure. These methods provide insight into atomic and molecular properties, which are normally difficult to obtain using standard spectroscopic methods and open up the possibility of designing chemical reactions induced by high pressure [10]. In the realm of methods capable of simulating molecules under pressure, a distinction can be made between the field of quantum mechanics (QM) and molecular dynamics (MD) simulations.

For QM methods, the extreme pressure polarization continuum model (XP‐PCM) [11, 12], the gaussians on surface tesserae simulate hydrostatic pressure (GOSTSHYP) [13], the generalized force‐modified potential energy surface (G‐FMPES) [14], the hydrostatic compression force field (HCFF) [15] and the extended hydrostatic compression force field (X‐HCFF) [16] shall briefly be mentioned. For a comprehensive overview of electronic structure simulation methods for high‐pressure chemistry, the reader is kindly referred to [9].

The XP‐PCM and GOSTSHYP methods both root in implicit solvation models [9]. As it is an extension of the polarizable continuum model (PCM) [17], in XP‐PCM the solvent is defined as a continuum with a uniform electron density ρ and dielectric permittivity ϵ, surrounding the solute in a molecule‐shaped cavity. The cavity is set up using the atomic van der Waals (VDW) radii of the molecule's atoms scaled by a factor or augmented with a probe radius to represent the solvent‐accessible surface (SAS) or the solvent‐excluded surface (SES) [18]. To apply pressure in XP‐PCM, the size of this cavity is reduced, and at the same time, ρ and ϵ are increased. This causes the continuum to come into closer contact with the electron density of the molecule, increasing pauli repulsion interactions and consequently inducing molecular compression [12, 19].

Like XP‐PCM, the GOSTSHYP method relies on a VDW surface‐shaped cavity surrounding the solute to exert the pressure. However, GOSTSHYP differs in the way the solvent is simulated by utilizing a uniform field of gaussian potentials on the cavity surface. These potentials result in a pressure being applied according to a force acting on a corresponding section of the cavity surface. In equilibrium the external forces are equal, but opposite in sign, to the restoring forces of the electron density of the molecule [13, 20].

The three remaining methods G‐FMPES, HCFF, and X‐HCFF, can be summarized as mechanochemical approaches for exerting pressure, which add external forces to the nuclear gradient during geometry optimizations. In G‐FMPES, the pressure is applied by modifying the potential energy surface of the system. This is done by applying a harmonic potential around the molecular system, leading to an external compression force $f_{\text{ext}}$ acting on all nuclei of the system. Each of these forces is applied in the direction of the geometric centroid of the molecule, whereby the magnitude of the force varies spatially depending on the distance to that centroid [14].

The HCFF method shows similarity to the G‐FMPES method, as again compression is achieved towards the molecular centroid. Furthermore, similarities with the implicit solvation methods can be identified, as the pressure is exerted via a molecule‐shaped cavity. The cavity allows the pressure to be applied as spatially varying forces f acting on the cavity area A. It should be noted that the pressure defined by the user ($P_{\text{guess}}$) is actually only an estimate, while the actual pressure ($P_{\mathrm{HCFF}}$) can be derived via the sum of all forces acting on the cavity surface. $P_{\mathrm{guess}}$ generally overestimates the pressure acting on the system [10, 16].

This paper focuses on the X‐HCFF method, which differs from its predecessor HCFF mainly in the direction of the forces used to induce compression. While in HCFF the forces act in the direction of the molecular centroid, in X‐HCFF forces act perpendicular to the molecular surface. This ensures that pressure is applied hydrostatically. Another advantage of X‐HCFF over previous methods is the user's ability to select the actual pressure rather than an estimate. As in the previously discussed cavity‐based methods GOSTSHYP and XP‐PCM, the size of the cavity can be scaled by a factor s. Typically, s takes values around 1.0–1.2 [16, 21] for the X‐HCFF, which are comparable to the commonly used scaling factors in XP‐PCM (∼0.7−1.3) [11, 19] and GOSTSHYP (∼1.0−1.5) [13, 20, 22], but s can also be increased to larger values if overlapping supramolecular VDW cavities are needed. We have observed that this scaling factor has a strong influence on the results of calculations in X‐HCFF, e.g., the critical pressure required to make a chemical reaction barrier‐free; however, this dependency on the scaling factor is undesirable. In the following, a rescaling formalism is presented that reduces this dependency for the X‐HCFF method. For this purpose, we first explain how forces act in X‐HCFF (Section 2.1) and how they can be rescaled (Section 2.2). Computational details are given in Section 3, after which we discuss the results of the rescaled X‐HCFF method (Section 4). Finally, conclusions are given in Section 5.

## 2 Theory

### 2.1 Traditional X‐HCFF

**FIGURE 1:**
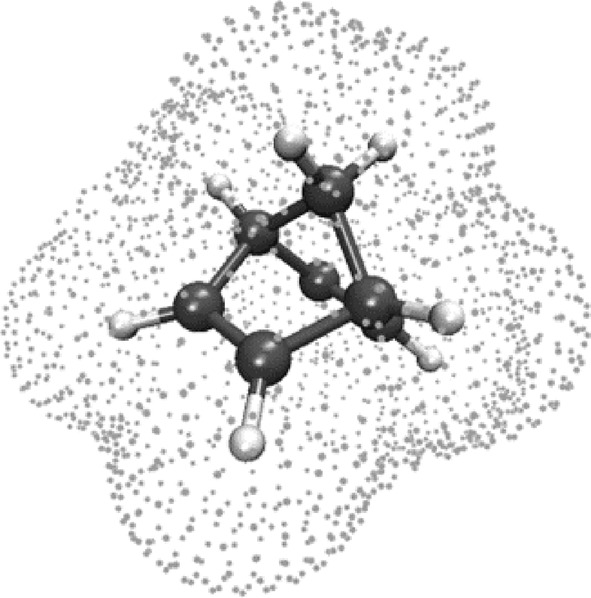
An exemplary tessellation field used in X‐HCFF for the pressure exerted on a chosen molecule.

The X‐HCFF method is a way to apply hydrostatic pressure in its mechanochemical definition to a molecule. In X‐HCFF, a tessellation field is generated using a lebedev discretization with a Switching/Gaussian (SwiG) algorithm [23, 24], which superimposes atom‐centered spheres with the appropriate VDW radius scaled by a factor s, resulting in a molecule‐shaped cavity (Figure 1).

As in PCM, scaling of the tessellation field is often required, as the pressure‐inducing atoms of the surrounding medium are generally found at a distance greater than the VDW radius of the individual atoms of the molecule. Using the classical definition of pressure P, which is defined as the normal component of the force f⊥ per area A, the forces acting in X‐HCFF can be obtained via

(1) $$f_{i,j}=-P\cdot A_{j}\cdot \frac{\left(r_{i}-r_{j}\right)}{\left|r_{i}-r_{j}\right|}.$$

Here $r_{i}$ and $r_{j}$ are the positions of the atom i and tessera j and $A_{j}$ is the area element of j (See Equation 4). The forces $f_{i}$ acting on each individual atom i can be calculated as the sum of forces $f_{i,j}$ acting from the direction of each tessera belonging to that atom.

(2) $$f_{i}=\overset{N_{\text{Tess}}(i)}{\underset{j}{\sum }}f_{i,j}$$

Since forces acting directly on opposite sides of each atom cancel each other, the resulting net force pushes the atoms closer toward each other, leading to a strictly hydrostatic compression of the molecular structure. The forces calculated according to Equation (2) are added to the nuclear gradient during a geometry optimization, while the tessellation field is updated on each optimization cycle. Convergence of a geometry optimization is achieved when the external forces from the pressure and the internal restoring forces of the molecule cancel. This type of compression allows simulations of pressure‐induced chemical reactions when more than one molecule is simulated and their tessellated cavities overlap [16]. Additionally, an analytical Hessian is available in X‐HCFF, allowing the investigation of pressure‐induced changes in vibrational frequencies [25]. Furthermore, the approach is able to simulate dynamic pressure in the context of ab initio molecular dynamics (AIMD) simulations [21].

### 2.2 Rescaled X‐HCFF

**FIGURE 2:**
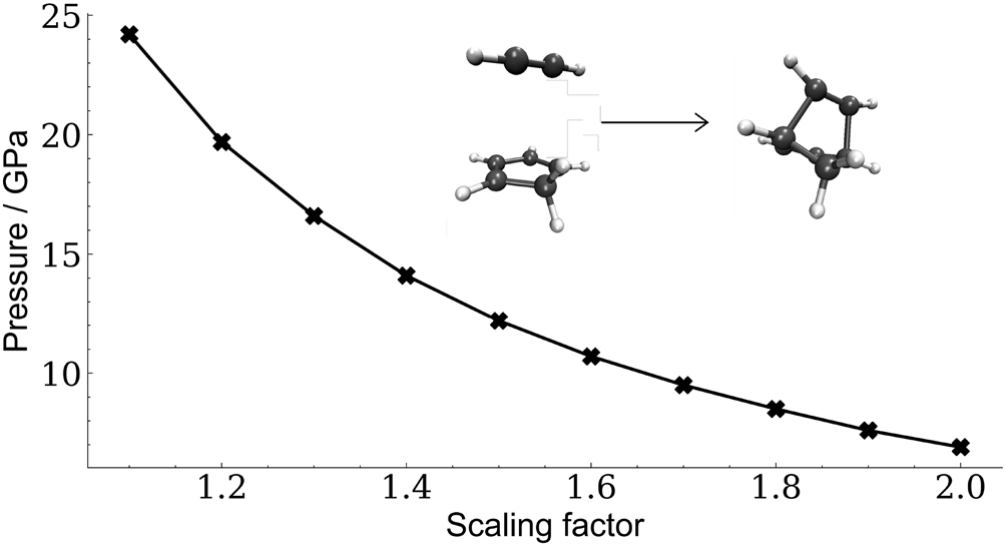
Pressure required to make the diels‐alder reaction between cyclopentadiene and acetylene barrier‐free, depending on the scaling factor of the VDW surface.

As can be seen in Figure 2 on the example of the diels‐alder reaction between cyclopentadiene and acetylene, the pressure required to make a chemical reaction barrier‐free depends on the scaling factor s of the VDW surface for the current implementation of X‐HCFF.

This observation can be explained by re‐examining the way X‐HCFF exerts pressure on molecules. The effect of the scaling factor s on the force $f_{i,j}$ experienced by a molecule can be described as

(3) $$f_{i,j}(s)=-P\cdot A_{j}(s)\cdot \frac{\left(r_{i}-r_{j}(s)\right)}{\left|r_{i}-r_{j}(s)\right|}$$

Since the fractional part of the equation is a vector normalization and the scaling factor can only take positive values, the effect of the scaling factor on $r_{j}(s)$ can be ignored. The dependency of the tessellated area $A_{j}(s)$ on the scaling factor s can be estimated using the following equation

(4) $$\begin{array}{cc} A_{j}(s) & \;=A_{i}(s)\cdot \zeta_{j}\cdot S_{j}(s)\\ & \;\approx A_{i}(s)\cdot \zeta_{j}\cdot S_{j}\\ & \;=A_{i}(1)\cdot s^{2}\cdot \zeta_{j}\cdot S_{j} \end{array}$$

with the lebedev integration weight $\zeta_{j}$ and the switching function $S_{j}$ [26], as the area $A_{i}$ is based on each tessellated atomic VDW sphere, which can be written as

(5) $$A_{i}(s)=4\cdot \pi \cdot {\left(R_{i}\cdot s\right)}^{2}$$

with the atomic VDW radius $R_{i}$ of atom i. This leads to increasing compressive forces on the atoms when s increases. However, the force can be made largely independent of s by rescaling it with a factor of $s^{-2}$.

(6) $${\hat{f}}_{i,j}=-P\cdot A_{j}\cdot \frac{1}{s^{2}}\cdot \frac{\left(r_{i}-r_{j}(s)\right)}{\left|r_{i}-r_{j}(s)\right|}$$

Similar rescaling is not possible for the other cavity‐based pressure methods such as GOSTSHYP and XP‐PCM. This is due to the intrinsic difference between these methods and X‐HCFF, as they rely on charge interaction between a dielectric continuum (XP‐PCM) or Gaussian potentials (GOSTSHYP) and the electronic density of the system for the pressure application. This removes the direct relationship between the size of the cavity surface and the applied forces, which is exploited in the rescaling approach for X‐HCFF presented here. Nevertheless, it should be noted that for these methods a change in the tessellated surface may also lead to a change in the pressure exerted on the system, making the choice of an adequate scaling factor very important. Methods to mitigate the problem of the ambiguous cavity choice in GOSTSHYP have recently been presented in literature [22, 27].

In this paper, we examine the performance of the rescaled X‐HCFF formalism, in which the external forces are calculated according to Equation (6).

## 3 Computational Details

All calculations were performed using a locally modified version of the Q‐Chem 6.0 program package [28]. For the X‐HCFF calculations, 302 tessellation points per atom and a pressure of 10 GPa were used unless stated differently. All calculations were performed at the B3LYP [29, 30, 31]/cc‐pVDZ [32] level of theory, with the addition of a DFT‐D3 correction [33]. The data generated for this publication was analyzed, processed, and visualized using Python 3.10 and Ovito [34].

## 4 Results and Discussion

To verify whether our rescaling formalism leads to scaling factor independence of X‐HCFF, we investigated a test set of 10 molecules: 1‐bromo‐2‐chloroethane, benzene, bromobenzene, chlorobenzene, ethane, fluoromethane, formamide, lithium hydride, methane, and sodium hydride. The molecules were chosen as they represent a variety of different common chemical structures and different atoms. It is important to cover a range of different VDW radii as well as different overlaps of atomic VDW spheres, since they lead to greatly differing tessellated VDW surfaces. As the evaluation method of choice, the gradient norms of X‐HCFF were chosen, as they represent the forces exerted according to Equation (6).

**FIGURE 3:**
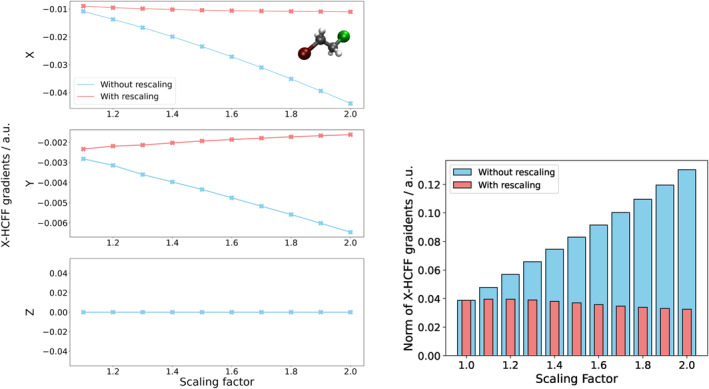
Left: X‐HCFF gradients acting on the chlorine atom of 1‐bromo‐2‐chloroethane in X, Y, and Z directions during the first cycle of an X‐HCFF geometry optimization at a pressure of 10 GPa as a function of the scaling factor. Right: Norm over all X‐HCFF gradient contributions for all atoms and spatial directions for 1‐bromo‐2‐chloroethane with and without rescaling.

As is exemplified in Figure 3 on 1‐bromo‐2‐chloroethane, the implemented rescaling formalism leads to the reduction of the dependency on the scaling factor in both the gradient norm and individual X‐HCFF gradient contributions. The remaining slope can mainly be explained by the way the tessellated surface is constructed in X‐HCFF. The implemented rescaling of the forces acting in X‐HCFF approximates the growth of the tessellated surface as the growth of the corresponding VDW radii (Equation (5)). While this approximation works quite well in most cases, areas in the tessellated surface where many VDW spheres overlap may lead to deviations from this trend. Fortunately, in many cases, this problem is negligible, as most of these atoms make barely any contribution to the surface area and therefore do not experience large amounts of force.

**FIGURE 4:**
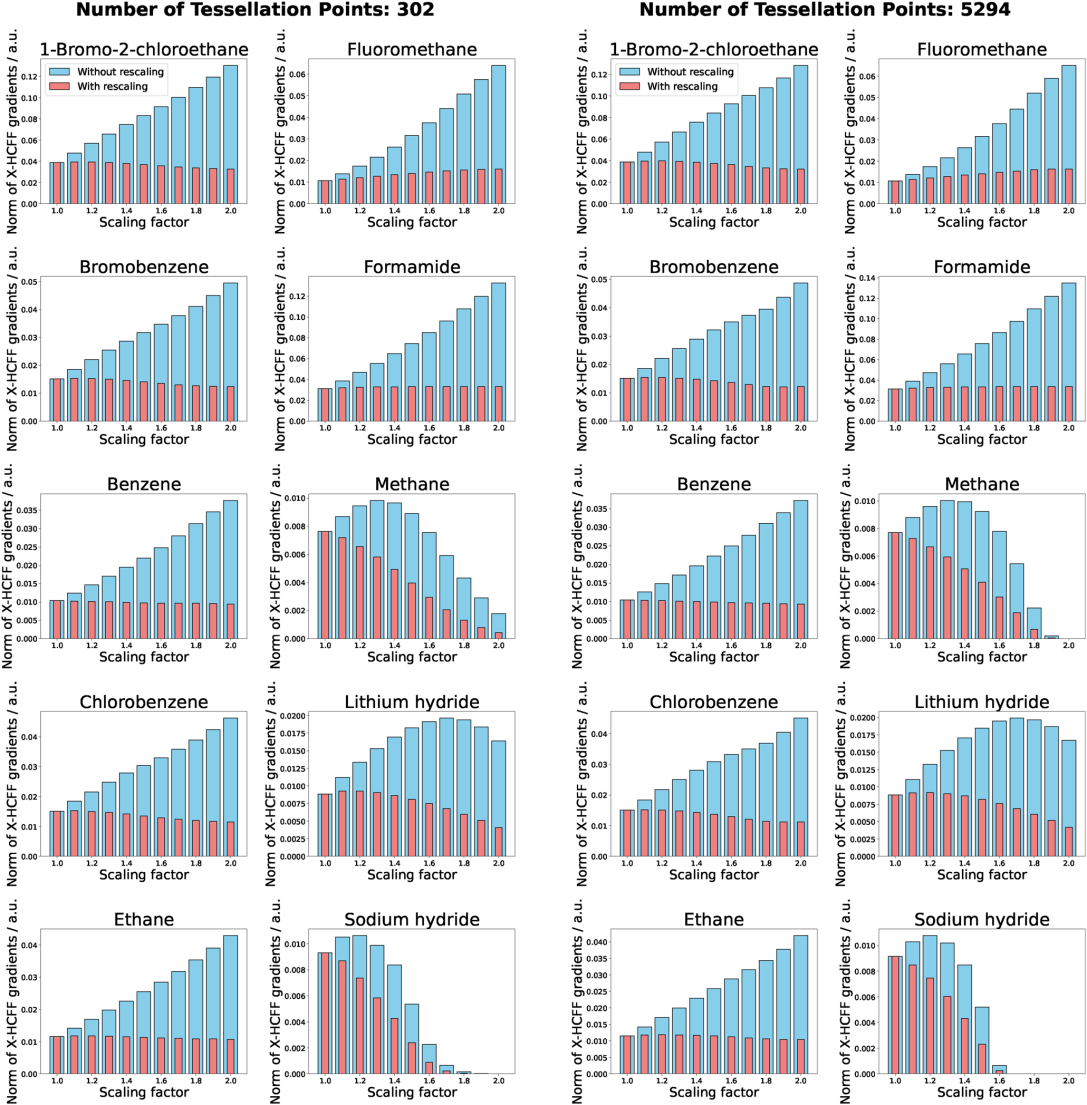
Dependency of the norm of the X‐HCFF gradients on the number of tessellation points per atom.

**FIGURE 5:**
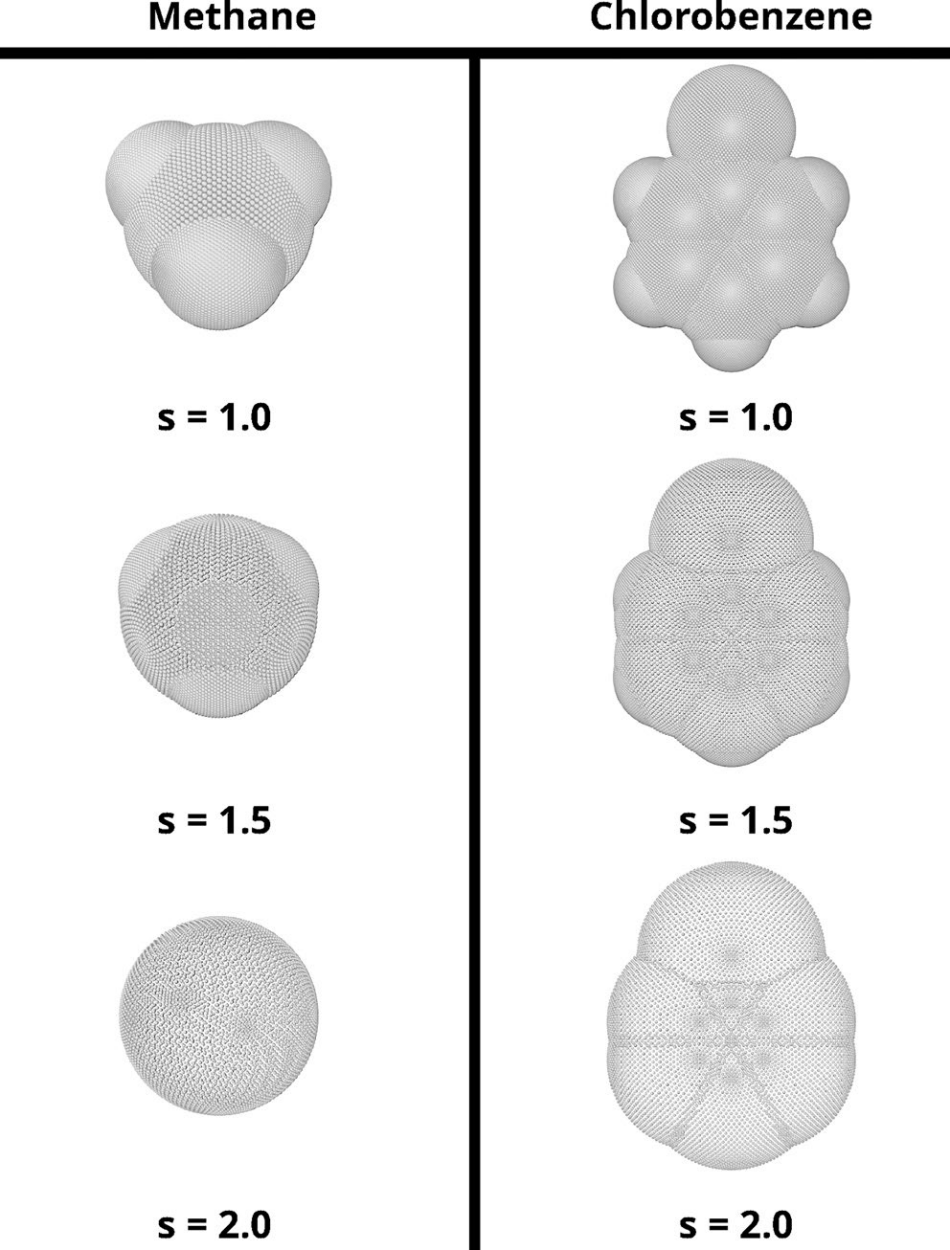
Visualization of the tessellated surface for methane and chlorobenzene using 5294 tessellation points and scaling factors of 1.0,1.5 and 2.0.

Since the surface overlap depends to a large extent on the surface structure, which is controlled by the number of tessellation points used, the effect of different amounts of tessellation points was additionally investigated. For this purpose, 110, 302, 350, 590, 1202, and 5294 tessellation points were used per atom, while the norm of all X‐HCFF gradient contributions was chosen as the evaluation method. As can be seen in Figures 4 and S1–S4, the X‐HCFF gradient norms are independent of the number of tessellation points chosen in almost all cases. However, the cases of methane and sodium hydride need to be further elaborated, as they show a remaining scaling factor dependency even after the rescaling, a dependency on the number of tessellation points used, and a disappearance of applied forces when large scaling factors are used. To investigate this behavior, the surface tesserae of methane and chlorobenzene were visualized using three different scaling factors (Figure 5).

It is evident that, at large scaling factors, the tessellated surface of the hydrogen atoms disappears due to the large VDW radius of the carbon atom. As mentioned in Section 2.1, directly opposing forces at the same atom cancel each other, explaining the absence of forces acting on the hydrogen atoms at high scaling factors (s≥2). We therefore propose not to use a scaling factor larger than 1.5 for molecules with strongly differing VDW radii. Additionally, the dependency on the number of tessellation points for these molecules can be explained by analyzing how the force cancellation works. Here, a larger number of tessellation points leads to a smoother tesserae surface and thus more surface vectors, increasing the probability of complete vector field cancellation as counter vectors are more likely to occur.

**FIGURE 6:**
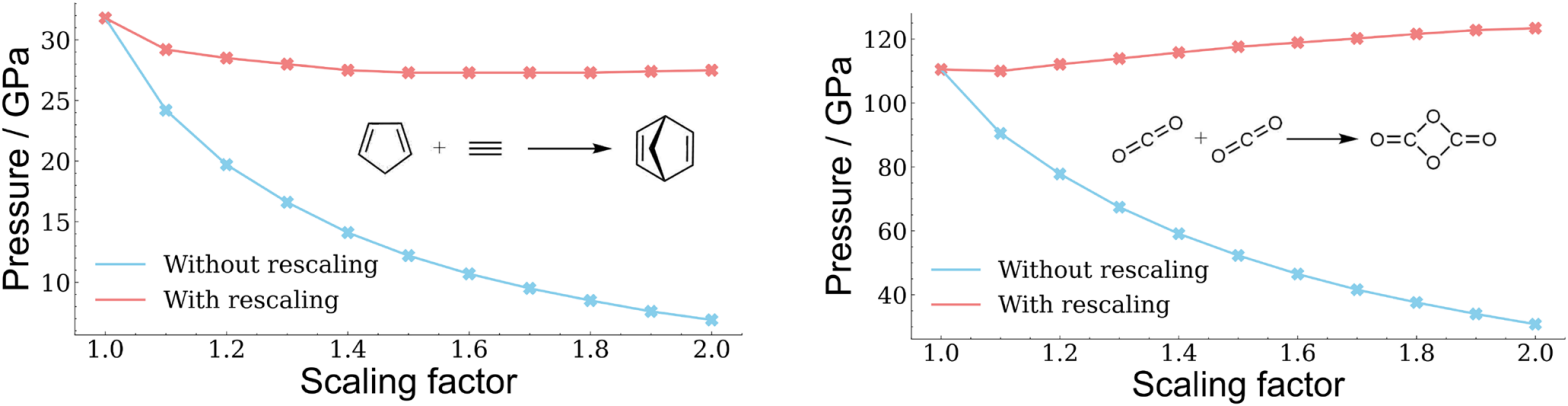
Comparison of the pressure required to make a reaction barrier‐free using X‐HCFF with and without rescaling. Left: diels‐alder reaction between cyclopentadiene and acetylene forming norbornadiene. Right: Dimerization of ${\text{CO}}_{2}$.

To test the applicability of the rescaled X‐HCFF formalism in a realistic scenario, two chemical reactions were verified, that is, the diels‐alder reaction of cyclopentadiene and acetylene [13] and the dimerization of carbon dioxide [35]. In both cases, rescaling causes a strong reduction of the dependency on the pressure required to make the reaction barrier‐free (Figure 6). In the case of the diels‐alder reaction, the remaining dependency can be explained by the change in the topology of the tessellated surface, where the overlap of the VDW cavities of the reactant molecules increases at higher scaling factors. This effect results in a decrease of the required pressure, which is particularly pronounced for smaller scaling factors, since counteracting forces are progressively reduced. The slight increase in pressure required in the case of the ${\text{CO}}_{2}$ dimerization can be explained by similar effects discussed previously for methane and the hydrides. Here again, an increase in the scaling factor results in an envelopment of the outer tessellation surface and therefore results in a more spherical surface structure, reducing the amount of forces acting on the system. Thus the pressure needed to enforce the reaction increases slightly.

**FIGURE 7:**
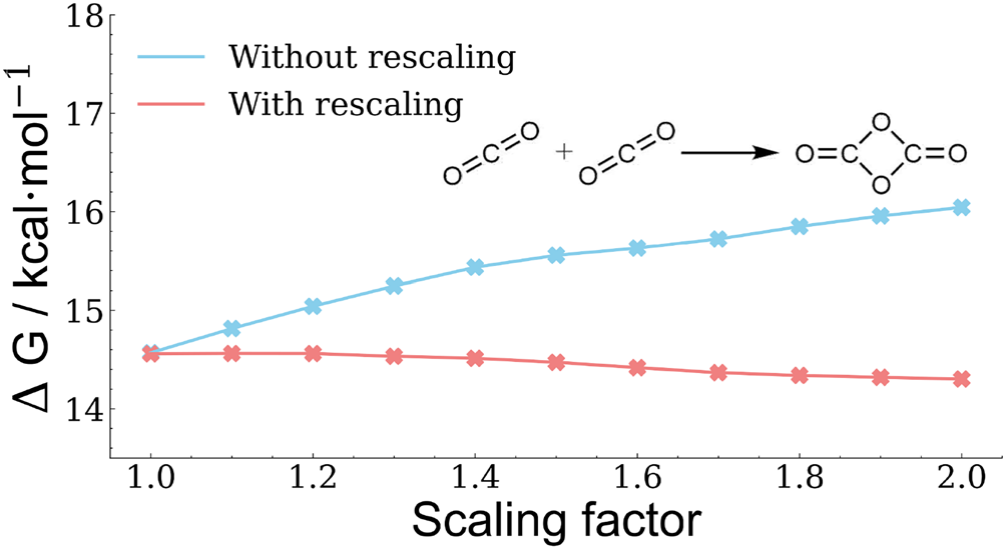
Comparison of the gibbs free energies for the dimerization of ${\text{CO}}_{2}$ using X‐HCFF with and without rescaling at a temperature of 298.15 K and a pressure of 125 GPa.

In addition to the pressure required for the ${\text{CO}}_{2}$ dimerization to become barrier‐free, the dependence of the gibbs free energies on the scaling factor was investigated as well (Figure 7).

Again, it can be seen that rescaling leads to a reduction in the scaling factor dependence. This is to be expected, as in the unscaled case the scaling factor is coupled to the force acting on the molecules. Larger scaling factors therefore lead to a stronger compression of the system's molecules and to a dependence of their gibbs free energies on the scaling factor, as compressing the molecules out of their pressure‐free state usually increases the system's energy. This consequently leads to a scaling factor dependence of the gibbs free energy if no rescaling is applied.

On top of these investigations, the use of different definitions of the VDW radii by Rowland and Taylor [36] and Bondi [37] were analyzed (see Supporting Information, Figures S5 and S6). This is important as the applied pressure strongly depends on the size of the VDW surface, which directly correlates with the size of the VDW radii of each individual atom. No clear advantage over the currently implemented VDW radius set (bondi VDW radii modified by rowland in the value of the hydrogen VDW radius) could be identified if a different set of VDW radii were implemented; hence, it is recommended to use the default set of VDW radii.

## 5 Conclusions

In this study, a rescaling formalism of the X‐HCFF high‐pressure simulation method was proposed that ensures compression forces consistent with the applied pressure. Rescaling of the forces was achieved by using the reciprocal square of the scaling factor s used to construct the tessellated molecular surface. This rescaling led to a significant reduction in the dependency of the X‐HCFF gradient on the scaling factor in all cases. Additionally, it was shown that the results hardly depend on the amount of tessellation points chosen for an X‐HCFF calculation. Furthermore, it was demonstrated that the rescaling formalism reduces the dependency of the pressure required to make a reaction barrier‐free on the scaling factor. Thus the presented formalism reduces the empiricism of the X‐HCFF model.

It is worth noting that the current analytical implementation of high‐pressure vibrational frequency analysis using X‐HCFF does not support the simultaneous application of rescaling [25]. Therefore, a semi‐numerical approach has to be used if high‐pressure frequency analyses are to be carried out using the rescaled X‐HCFF method.

## Supporting information




**Data S1.** Supporting Information.

## Data Availability

The data that supports the findings of this study are available in the Supporting Information of this article.
