# Supplementary material for: Achieving Pressure Consistency in Mechanochemical Simulations of Chemical Reactions Under Pressure
Source: J Comput Chem. 2025 Jan 26;46(3):e70024. doi: 10.1002/jcc.70024 (PMC11771609; doi:10.1002/jcc.70024)
Supplement: Supplementary file 1 — Data S1. Supporting Information. [file JCC-46-0-s001.pdf]

SUPPORTING INFORMATION FOR: "ACHIEVING PRESSURE CONSISTENCY IN MECHANOCHEMICAL SIMULATIONS OF CHEMICAL REACTIONS UNDER PRESSURE"

Jonas Bentrup[1], Rahel Weiß[1], Felix Zeller[1], Tim Neudecker[1,2,3]
[1]University of Bremen, Institute for Physical and Theoretical Chemistry, Leobener Straße 6, D-28359 Bremen, Germany
[2]Bremen Center for Computational Materials Science, University of Bremen, Am Fallturm 1, D-28359 Bremen, Germany
[3]MAPEX Center for Materials and Processes, University of Bremen, Bibliothekstraße 1, D-28359 Bremen, Germany

List of Figures

1 Dependency of the norm of the X-HCFF gradients on the scaling factor using 110 tessellation points for all atoms and a pressure of 10 GPa. 2
2 Dependency of the norm of the X-HCFF gradients on the scaling factor using 350 tessellation points for all atoms and a pressure of 10 GPa. 3
3 Dependency of the norm of the X-HCFF gradients on the scaling factor using 590 tessellation points for all atoms and a pressure of 10 GPa. 4
4 Dependency of the norm of the X-HCFF gradients on the scaling factor using 1202 tessellation points for all atoms and a pressure of 10 GPa. 5
5 Dependency of the norm of the X-HCFF gradients on the selected set of VDW radii. For these calculations, the definition of VDW radii according to Bondi using 302 tessellation points for all atoms and a pressure of 10 GPa was chosen. 6
6 Dependency of the norm of the X-HCFF gradients on the selected set of VDW radii. For these calculations, the definition of VDW radii according to Rowland using 302 tessellation points for all atoms and a pressure of 10 GPa was chosen. 7

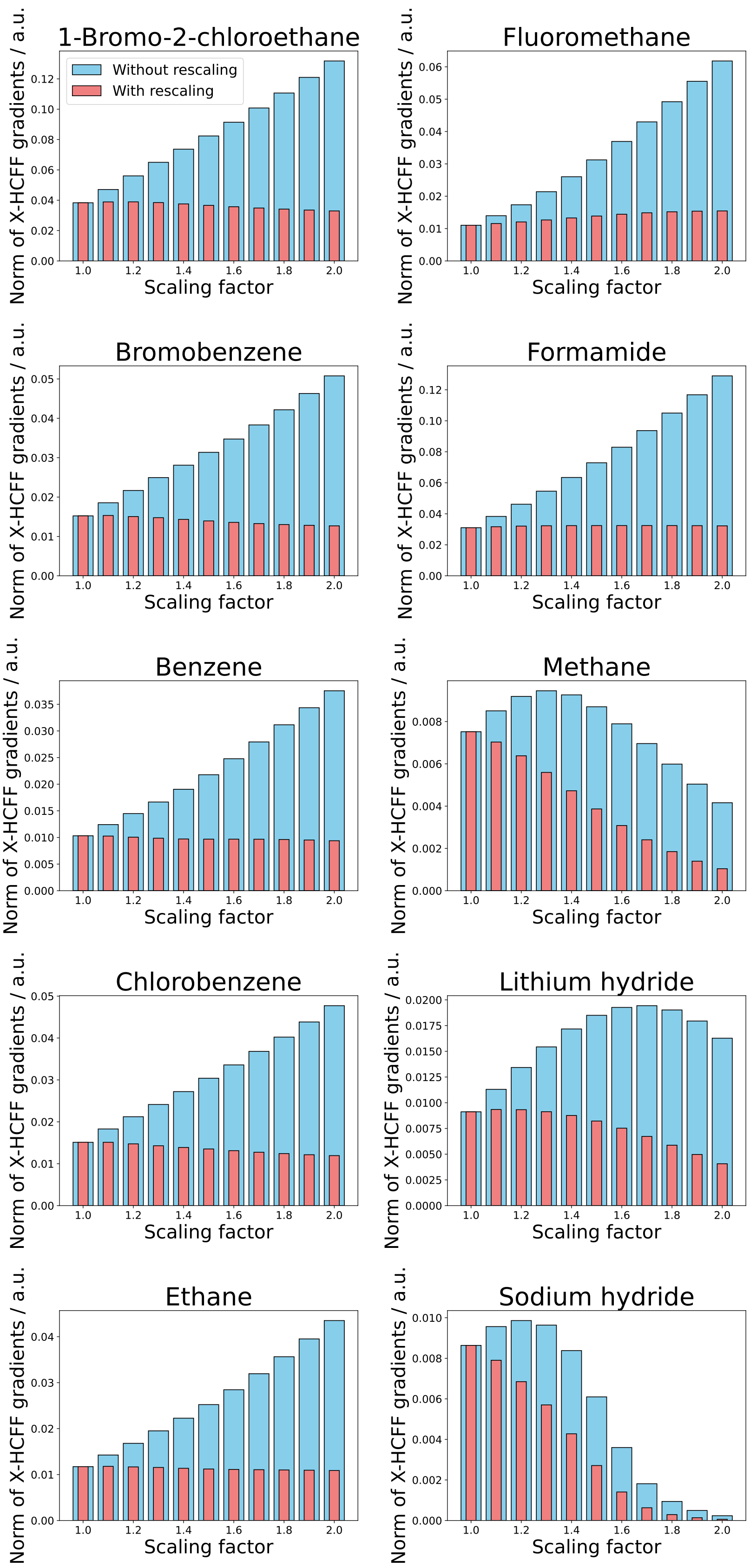

Figure S 1: Dependency of the norm of the X-HCFF gradients on the scaling factor using 110 tessellation points for all atoms and a pressure of 10 GPa.

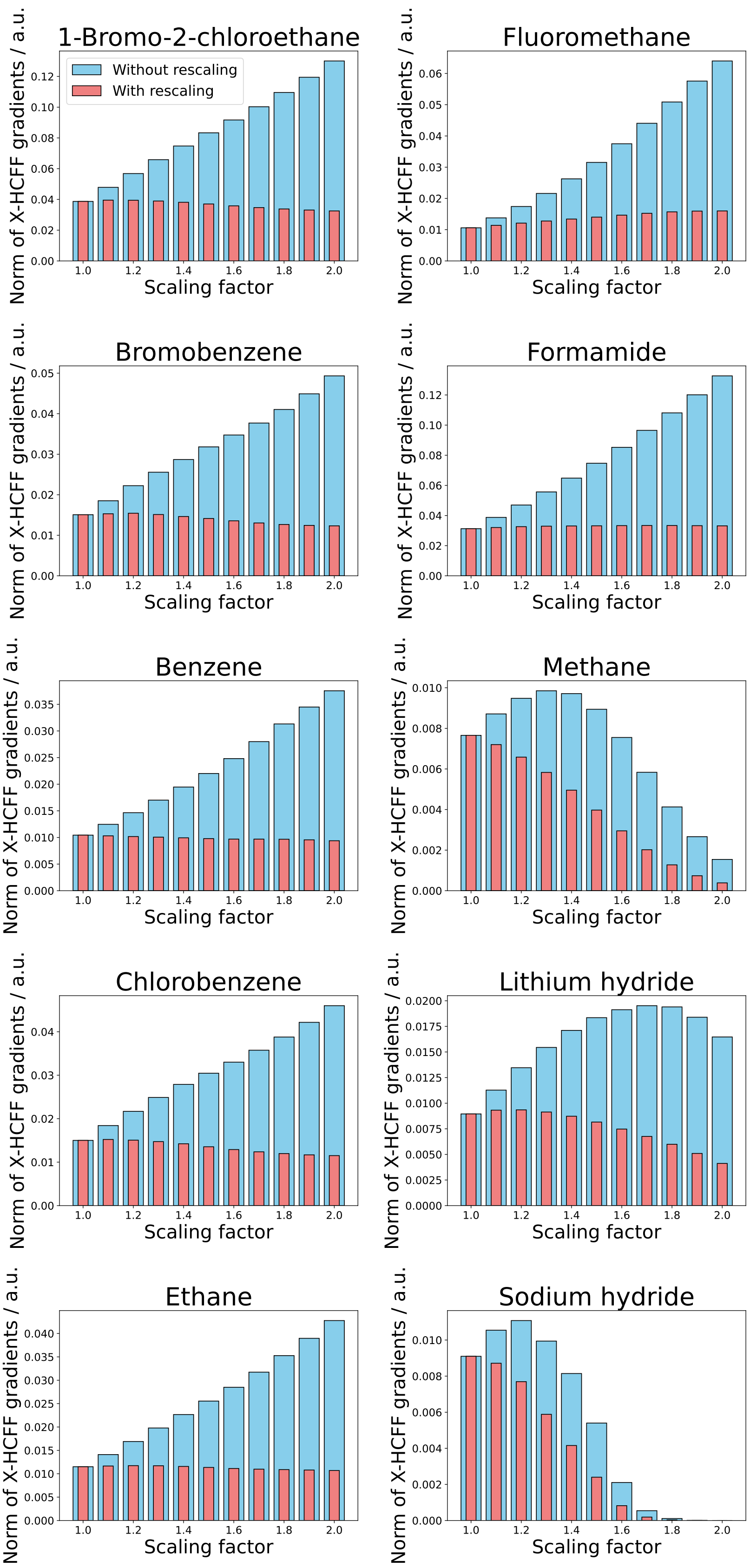

Figure S 2: Dependency of the norm of the X-HCFF gradients on the scaling factor using 350 tessellation points for all atoms and a pressure of 10 GPa.

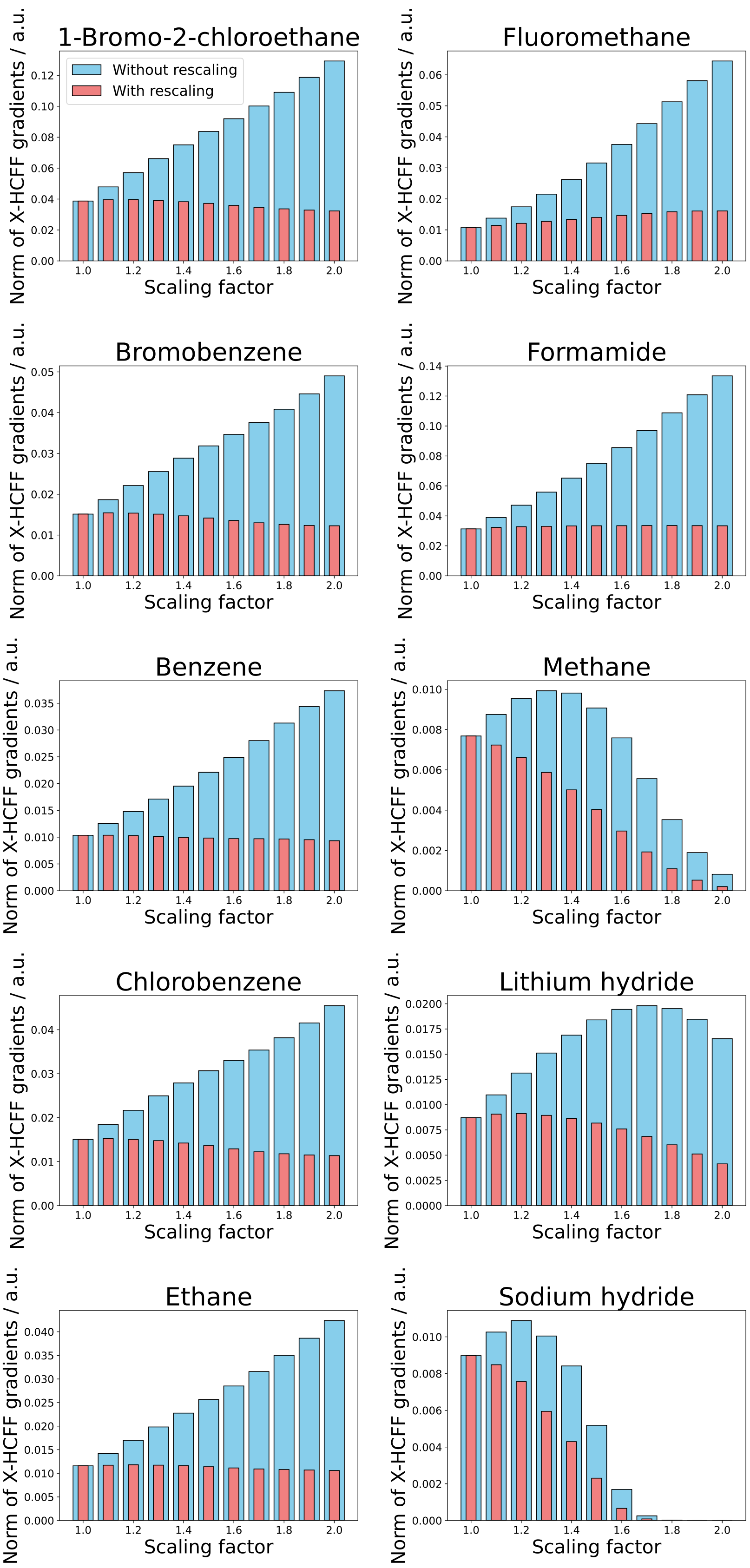

Figure S 3: Dependency of the norm of the X-HCFF gradients on the scaling factor using 590 tessellation points for all atoms and a pressure of 10 GPa.

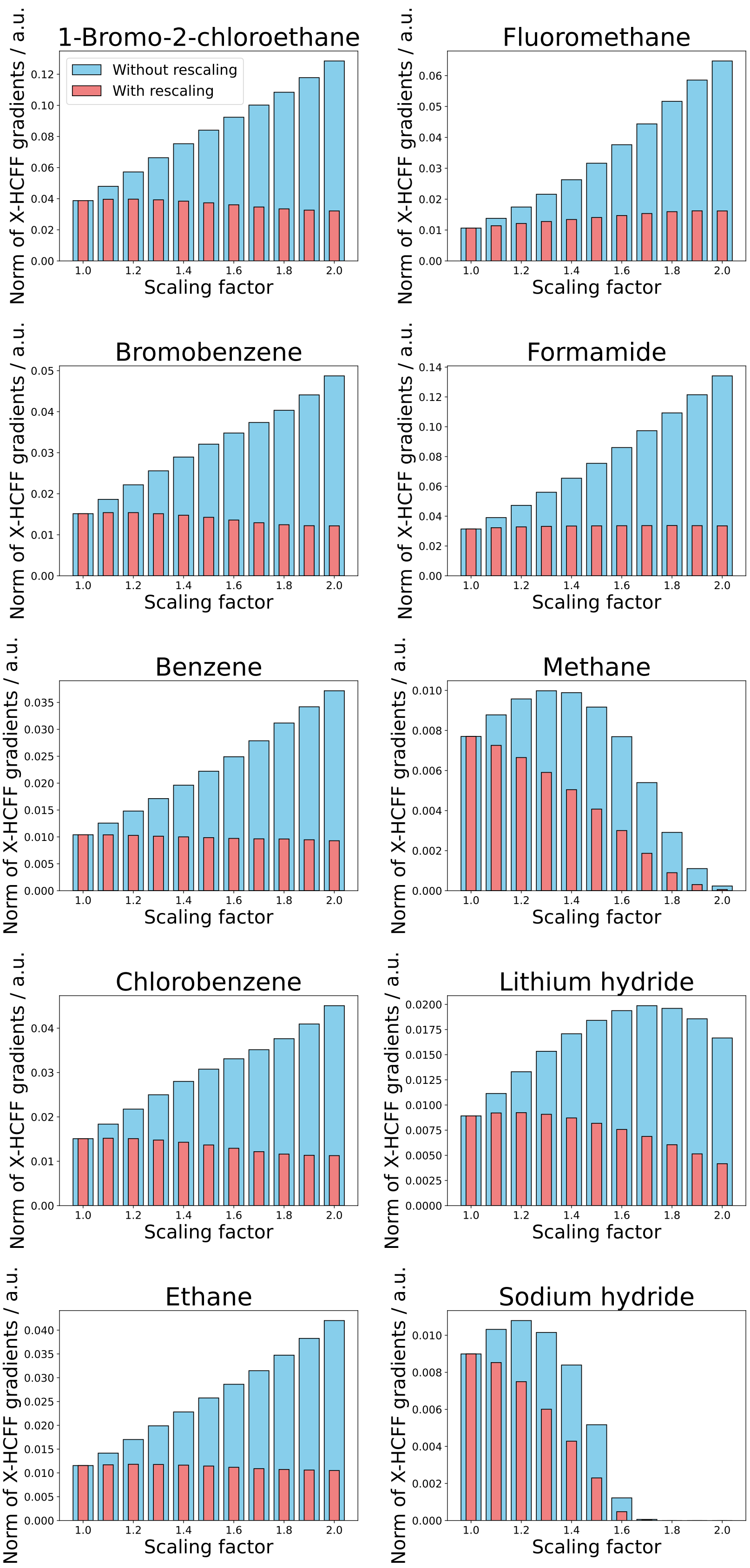

Figure S 4: Dependency of the norm of the X-HCFF gradients on the scaling factor using 1202 tessellation points for all atoms and a pressure of 10 GPa.

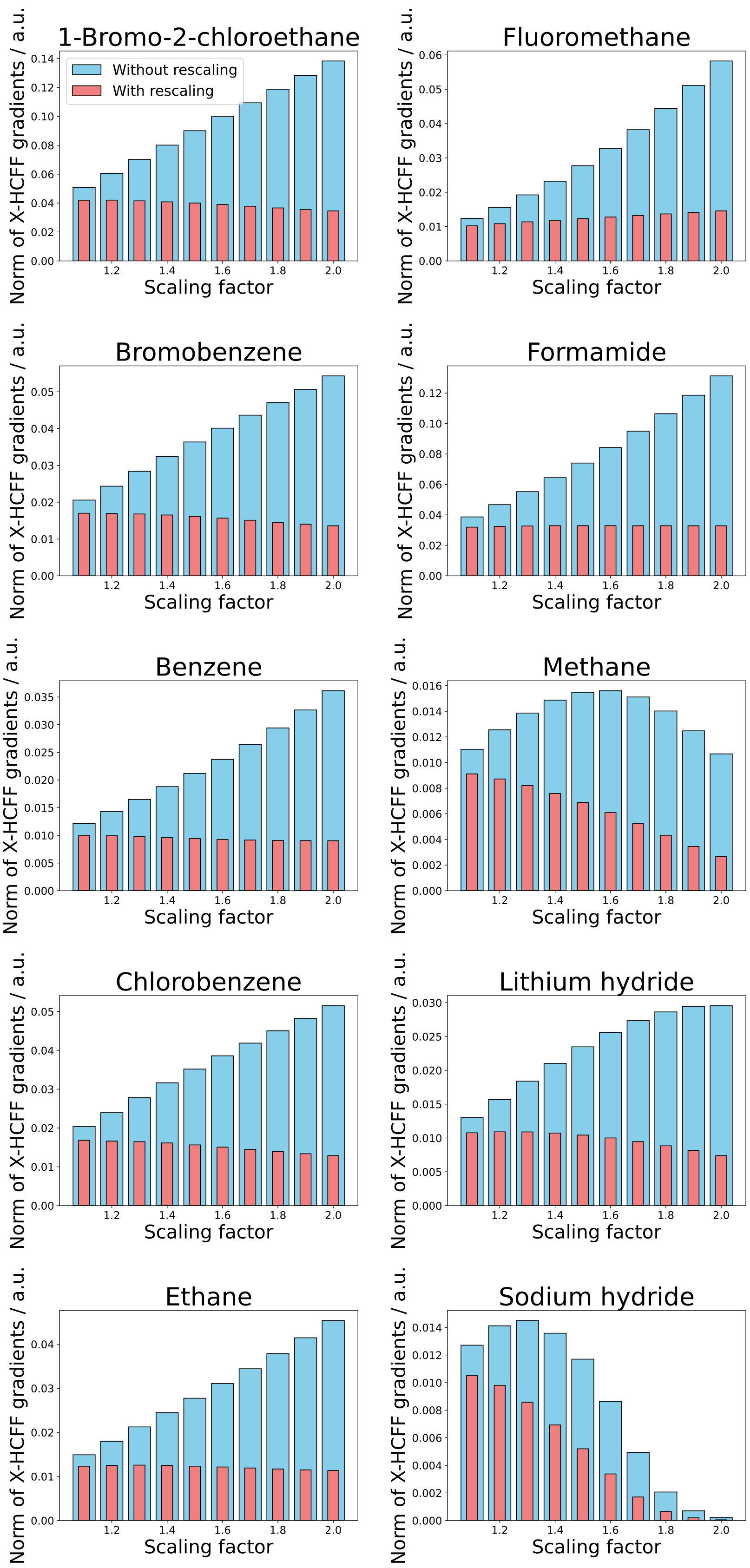

Figure S 5: Dependency of the norm of the X-HCFF gradients on the selected set of VDW radii. For these calculations, the definition of VDW radii according to Bondi using 302 tessellation points for all atoms and a pressure of 10 GPa was chosen.

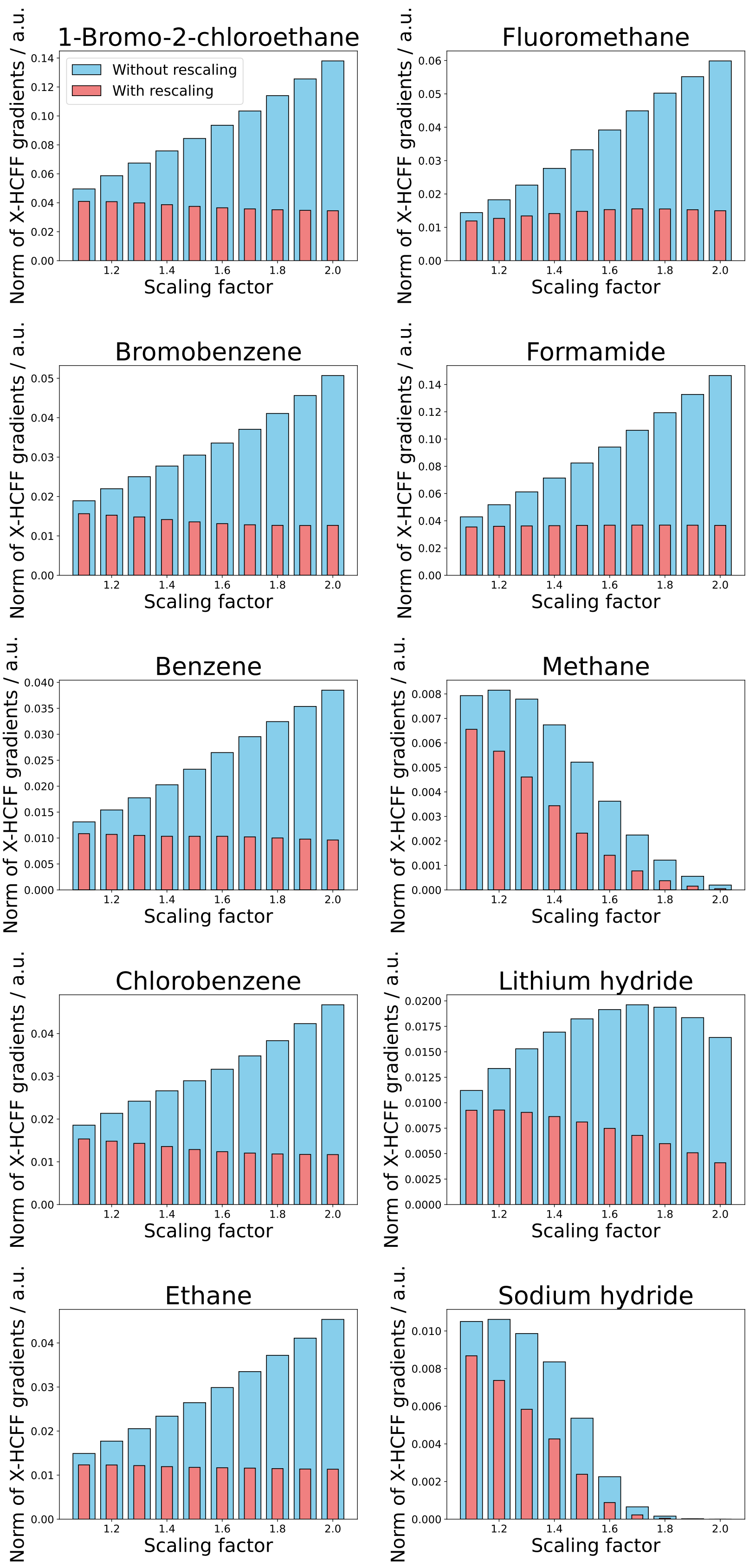

Figure S 6: Dependency of the norm of the X-HCFF gradients on the selected set of VDW radii. For these calculations, the definition of VDW radii according to Rowland using 302 tessellation points for all atoms and a pressure of 10 GPa was chosen.
